# Small junction, big problems: Neuromuscular junction pathology in mouse models of amyotrophic lateral sclerosis (ALS)

**DOI:** 10.1111/joa.13463

**Published:** 2021-06-07

**Authors:** Abrar Alhindi, Ines Boehm, Helena Chaytow

**Affiliations:** ^1^ Edinburgh Medical School Biomedical Sciences University of Edinburgh Edinburgh UK; ^2^ Department of Anatomy Faculty of Medicine King Abdulaziz University Jeddah Saudi Arabia; ^3^ Euan MacDonald Centre for Motor Neurone Disease Research University of Edinburgh Edinburgh UK

**Keywords:** C9orf72, denervation, dying‐back, FUS, NMJ, SOD1, TDP‐43

## Abstract

Amyotrophic lateral sclerosis (ALS) is a motor neuron disease with an extremely heterogeneous clinical and genetic phenotype. In our efforts to find therapies for ALS, the scientific community has developed a plethora of mouse models, each with their own benefits and drawbacks. The peripheral nervous system, specifically the neuromuscular junction (NMJ), is known to be affected in ALS patients and shows marked dysfunction across mouse models. Evidence of pathology at the NMJ includes denervated NMJs, changes in endplate size and loss of terminal Schwann cells. This review compares the temporal disease progression with severity of disease at the NMJ in mouse models with the most commonly mutated genes in ALS patients (*SOD1, C9ORF72, TARDBP* and *FUS*). Despite variability, early NMJ dysfunction seems to be a common factor in models with *SOD1, TARDBP* and *FUS* mutations, while *C9ORF72* models do not appear to follow the same pattern of pathology. Further work into determining the timing of NMJ pathology, particularly in newer ALS mouse models, will confirm its pivotal role in ALS pathogenesis and therefore highlight the NMJ as a potential therapeutic target.

## Graphical abstract

Summary of neuromuscular junction (NMJ) changes across ALS mouse models. ALS mouse models show different levels of NMJ pathology depending on gene targets: SOD1, TDP‐43 and FUS models all show initial NMJ dysfunction prior to motor neuron death (arrows), supporting a ‘dying‐back’ theory, while C9orf72 NMJ pathology is less clear. Terminal Schwann cells (tSCs) are lost in the SOD1 model. Dark red regions on the muscle fibre represent the endplate, which is smaller and fragmented in some C9orf72, TDP‐43 and FUS models.

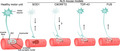

## 1 INTRODUCTION

Amyotrophic lateral sclerosis (ALS) is an adult‐onset motor neuron disease characterised by the specific cell death of both upper and lower motor neurons. This leads to a failure in neuromuscular transmission, resulting in progressive muscle weakness leading to paralysis. Ultimately, ALS is fatal usually within 5 years of diagnosis as a result of respiratory failure (Hardiman et al., 2017). The vast majority of ALS cases are considered sporadic (sALS), where both genetic and environmental factors contribute to disease pathogenesis, and only 10% of cases show a family history (familial ALS; fALS). The most commonly mutated genes in ALS patients are *SOD1, C9ORF72, TARDBP* and *FUS*, accounting for over half of fALS patients but less than 10% of sALS patients (Zou et al., 2017). Rare mutations in dozens of other genes have been associated with ALS, including *UBQLN2, SQSTM1, TBK1, VAPB* and *VCP* (Kim et al., 2020). Through studying the overlapping mechanisms of these genes and their proteins, pathways such as RNA processing, protein trafficking and axonal dysfunction have been implicated in ALS disease progression (Kim et al., 2020). When ALS was first described in 1865 by Charcot, the major pathological trait was the specific cell death of anterior horn cells in the spinal cord (Charcot, 1865). Since then, it has become clear that the distal end of the motor neuron, the neuromuscular junction, plays an important role in ALS pathology.

### 1.1 Neuromuscular junction (NMJ) dysfunction in health and disease

**FIGURE 1:**
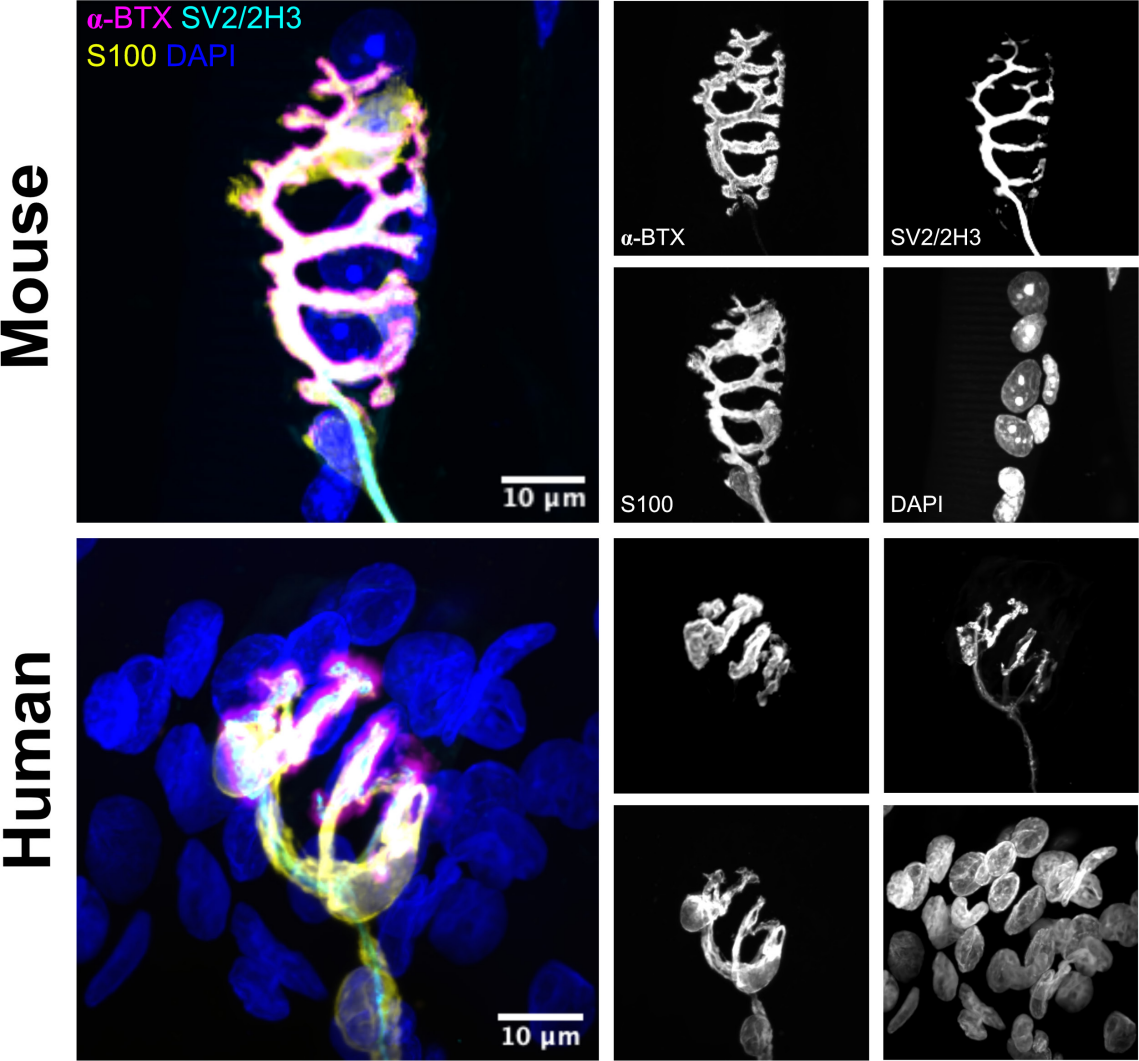
Representative confocal images show the marked difference of NMJ morphology between mouse (top panels) and human (lower panels) in *peroneus brevis* muscles (images taken from data set published in Alhindi et al., 2021). Mouse NMJs are pretzel‐shaped and larger in size, while human NMJs are smaller and have fragmented (nummular‐shaped) endplates. Images have been pseudo‐coloured for display purposes. The panels to the left show a composite image of acetylcholine receptors (magenta) labelled by α‐BTX (alpha‐bungarotoxin), axon and nerve terminals (cyan) labelled by anti‐SV2/2H3, terminal Schwann cells (yellow) labelled by anti‐S100 and nuclei (blue) labelled by DAPI. The panels to the right depict the respective channels in grey scale. Scale bar = 10 µm

The NMJ is the name of the synapse between a motor neuron and the muscle fibre that it innervates. It is critically important that the messages from the motor neuron are efficiently and reliably passed on to the muscle fibre to produce the intended movement, and so NMJ stability is crucial. A healthy NMJ, pictured in Figure 1, consists of a nerve terminal from a motor neuron, the pre‐synaptic side (labelled with SV2/2H3 in Figure 1), covering the ‘endplate’ on the post‐synaptic side, a cluster of invaginations in the muscle fibre membrane covered in acetylcholine receptors (AChRs, labelled with α‐BTX in Figure 1), (Ruff, 2003). When an action potential reaches the nerve terminal, the neurotransmitter acetylcholine is released from synaptic vesicles into the synaptic cleft, activating AChRs and triggering contraction of the muscle fibre. The structure and function of the NMJ are supported by the terminal Schwann cell (tSC; labelled with S100 in Figure 1), a non‐myelinating glial cell located at the NMJ (Santosa et al., 2018). The clearest indicator of NMJ dysfunction is a denervated, or partially denervated, NMJ, where the pre‐synaptic nerve terminal no longer covers the post‐synaptic endplate. This type of denervation is often quantified to assess the degree of NMJ dysfunction in disease models (Liu et al., 2016; Rocha et al., 2013).

Electrophysiological measurements commonly used to assess the function of a synapse include the miniature endplate potential (MEPP), quantal content and mean quantal stores. The MEPP is the change in postsynaptic membrane potential caused by the spontaneous release of a single ‘quantum’ (a single synaptic vesicle which contains about 5–10,000 molecules of acetylcholine) in the absence of a nerve impulse, which is not sufficient to generate a muscle action potential (Howard, 2003). These quanta are stored in synaptic vesicles ready for immediate release. An action potential in the motor neuron leads to the release of 50–100 quanta of acetylcholine from the quantal stores into the synaptic cleft in rodents (20–50 in humans), in a process called quantal release (Howard, 2003). Normally, the number of quanta released is more than that needed for the generation of a muscle action potential, even at high frequency activity (10–100 Hz), a feature called safety factor (Wood & Slater, 2001). The EPP amplitude and quantal content are, therefore, useful measurements of synaptic function and effectiveness of transmission. Reduced quantal content or quantal stores would lead to less acetylcholine being released per action potential, which would then affect the strength of the muscle contraction as indicated by a reduced MEPP amplitude. Evidence from multiple models of neurodegeneration indicates that NMJs showing degeneration morphologically, such as partial denervation, reduced endplate size or neurofilament accumulation, retain some synaptic transmission due to the safety factor but this slowly reduces in efficacy over time (Gillingwater et al., 2002; Kong et al., 2009).

Further markers of dysfunction at the NMJ include reduced numbers of synaptic vesicles and abnormal pre‐synaptic mitochondria (So et al., 2018), or smaller (Picchiarelli et al., 2019) or fragmented endplates post‐synaptically (Ditsworth et al., 2017) as well as abnormal tSCs, either through changes in morphology or loss of tSCs altogether (Santosa et al., 2018). The ‘dying‐back’ hypothesis describes an initiation of degeneration at the NMJ or distal nerve terminal, followed by retrograde axonal degeneration and successive motor neuron death (Coleman, 2005). Conversely, the ‘dying‐forward’ hypothesis suggests that dysfunction in the motor neuron cell body itself is the starting point, with successive anterograde axonal degeneration and NMJ dysfunction (Geevasinga et al., 2016). Teasing apart the order of these events is critical in order to understand the sequence of ALS pathology, and therefore aid better drug discovery.

### 1.2 NMJs in human ALS patients

There are many challenges in studying the human NMJ, including sample collection, appropriate controls for age, sex or disease duration, and linking to complete medical and family histories of disease. Disease progression assessment is almost impossible, as sampling pre‐symptomatically is not available in such an apparently idiopathic disease, and multiple sample collections across time points post‐diagnosis are difficult. However, despite these challenges and limitations, there have been notable successes. A combination of muscle biopsies, post‐mortem analyses and electrophysiological assessment has shown that NMJ dysfunction plays a critical, early role in ALS disease progression.

An early study using simple staining techniques for AChRs on intercostal muscle biopsies found more fragmented endplates in ALS patients compared to controls, which did not correlate with clinical progression (Bjornskov et al., 1975). The development of a double stain for nerve terminals and endplates highlighted evidence of denervation as well as the fragmented endplates (Bjornskov et al., 1984). Analysis using electron microscopy further described a trend towards smaller nerve terminal and endplate areas in ALS patients in biopsies from the biceps, as well as a reduced number of observed mitochondria at the nerve terminal (Tsujihata et al., 1984). This was replicated in analysis of laryngeal muscles from ALS patients, which identified multiple pathological changes including denervation and flattened synaptic clefts, and even found some potential evidence of regeneration at the NMJ with small nerve terminals over distorted endplates. However, it is difficult to distinguish whether the described morphology is part of the process of denervation or a sign of active reinnervation (Yoshihara et al., 1998). Further molecular characteristics of pathology at the NMJ in ALS patients include a change in AChR subunit expression (Palma et al., 2016), increased Nogo‐A expression (Bruneteau et al., 2015) and changes in pre‐synaptic voltage‐gated calcium channel subunit expression (Day et al., 1997). The exception to this rule of denervation in ALS muscle is the group of extraocular muscles, which have intrinsic differences to other skeletal muscles and are spared in ALS pathology (Nijssen et al., 2017).

Intracellular recordings from ALS patient biopsies showed decreased MEPP amplitude, reduced quantal content and reduced quantal stores (Maselli et al., 1993). The strongest evidence that this NMJ dysfunction occurs prior to motor neuron cell death came from the autopsy of an ALS patient who died unexpectedly following 6 months of muscle weakness (Fischer et al., 2004). Clinical electromyographic (EMG) tests showed evidence of denervation in both upper and lower limb muscle groups in this patient 2 weeks prior to their death, and post‐mortem analysis of the spinal cord and ventral root showed no changes in motor neuron number (Fischer et al., 2004). However, comparison of NMJ morphology from muscle biopsies and correlation with EMG variables showed no relationship between the degree of change in the EMG with morphological characteristics (Bruneteau et al., 2015).

TSCs have also been reported to be altered in human ALS patients. They showed reduced staining for both S100b, a well‐known tSC marker, and p75^NTR^, a neurotrophin receptor expressed in tSCs during development and in the case of nerve injury, in post‐mortem limb muscles (*tibialis anterior, vastus lateralis, biceps brachii*) (Liu et al., 2013). However, in extraocular muscles, S100b labelling was absent, but p75^NTR^ was still present, indicating a downregulation of S100b protein rather than tSC loss (Liu et al., 2013). Others have found that tSCs, labelled by S100, showed deposition of complement activation products in the intercostal muscles (Bahia El Idrissi et al., 2016) and abnormal cytoplasmic processes that extend into the synaptic cleft between nerve terminals and AChRs in the deltoid, and *anconeus* muscles (Bruneteau et al., 2015).

The culmination of this body of evidence of NMJ dysfunction in ALS patients shows morphological, molecular and functional changes at the NMJ prior to motor neuron cell death, thereby indicating a ‘dying‐back’ pathology. To further investigate the role of NMJ dysfunction in ALS disease progression, we turn to mouse models. However, modelling ALS in animal models is fraught with difficulties. Firstly, when discussing the NMJ, the species differences between mouse and human must be considered. Human NMJs maintain a smaller, fragmented structure throughout adulthood, compared to the classic ‘pretzel‐like’ structure seen in mice (Jones et al., 2017). Human NMJs are also associated with smaller tSCs that are morphologically different to mouse tSCs (Alhindi et al., 2021), as highlighted in Figure 1. While mouse models showing NMJ dysfunction may show increased fragmentation patterns, it is difficult to know how this translates to human NMJs. Secondly, the heterogeneity of genetic and environmental factors prevents any mouse model from properly replicating disease progression. Instead, each of the *SOD1, C9ORF72, TARDBP* and *FUS* genes as well as others have been targeted to create mouse models that to a greater or lesser extent mimic phenotypes seen in ALS patients. The most common mutation, the expansion repeat in *C9ORF72*, is logistically very difficult to produce in mice due to the large size of the insertion. Other models such as the SOD1^G93A^ and TDP‐43^Q331K^ mice reproduce mutations that are very rare in patient populations. These factors will account for the variability found across models and difficulties in translation to ALS patients. In this review, we will discuss NMJ dysfunction in the most common mouse models of ALS, and whether denervation occurs at the symptomatic stages of the model, where the mouse shows motor behaviour dysfunction, or presymptomatically, before motor phenotypes occur.

## 2 SOD1

Mutations in the Cu/Zn‐binding superoxide dismutase (*SOD1*) gene were the first to be associated with ALS, accounting for around 20% of fALS patients (Rosen et al., 1993; Zou et al., 2017). While over 150 mutations have been annotated to date, only a few of them have been shown to cause ALS (Renton et al., 2014). SOD1 is a cytosolic enzyme that catalyses reactive free radicals and mutations lead to a gain of cytotoxic function as a result of abnormal aggregation of misfolded SOD1 protein in motor neurons and muscles (Turner et al., 2003).

Distinct *SOD1* mutations have been associated with different ALS phenotypes. For example, patients homozygous for the D90A mutation have a spinal onset disease with a slow rate of progression, where it takes up to 10 years for the disease to affect the respiratory muscles (Renton et al., 2014). However, patients heterozygous for D90A have a bulbar, upper limb or lower limb onset, that progresses rapidly (Li & Wu, 2016). Similarly, G93A, A4V, H43R, L84V, G85R and N86S mutations are associated with an aggressive disease course and shorter survival while G93C and H46R mutations are associated with longer life expectancies (Juneja et al., 1997). Here, we will focus our analyses on NMJ changes occurring in the SOD1^G93A^ and SOD1^G37R^ mouse models of ALS.

### 2.1 NMJ changes in the SOD1^G93A^ mouse model

**FIGURE 2:**
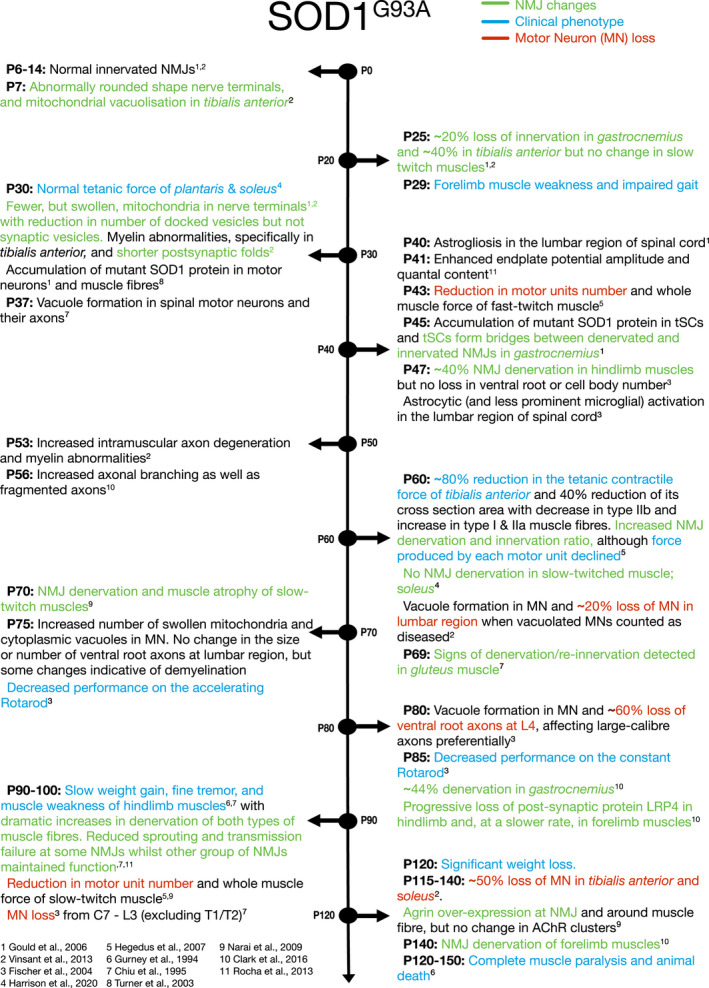
Timeline of NMJ denervation in the SOD1^G93A^ mouse model. Blue descriptions indicate ‘clinical’ phenotypes. Green descriptions indicate NMJ changes. Red descriptions indicate motor neuron (MN) loss

The SOD1^G93A^ mouse, which expresses mutant human SOD1 with a glycine‐to‐alanine substitution at position 93, is the most commonly used ALS model to study disease pathophysiology. The animal develops progressive motor weakness, muscle atrophy and paralysis similar to ALS patients with a median survival of around 120 days (Gurney et al., 1994). Several studies have extensively reported NMJ changes in this model. In general, pathology affects the fast‐twitch muscle fibres before slow‐twitch counterparts and starts in the hindlimb muscles before the forelimb, with the earliest changes appearing in nerve terminals prior to any changes in either the post‐synaptic region or tSCs, as shown in Figure 2 (Clark et al., 2016; Schaefer et al., 2005).

Evidence for NMJ degeneration in SOD1^G93A^ mice begins at very early, pre‐symptomatic stages. At P7‐P14, while NMJs are still intact, investigations in the *tibialis anterior* (TA) and *gastrocnemius* (GC) of SOD1^G93A^ mice found that the nerve terminals had a more rounded shape compared to their normal elongated appearance, with abnormal mitochondria in pre‐synaptic terminals of the TA (Gould et al., 2006; Vinsant et al., 2013). The first evidence of NMJ denervation in the SOD1^G93A^ mouse is at P14‐P30 in fast‐twitch muscles of asymptomatic mice. By P30, 20% of GC (Gould et al., 2006) and 40% of the TA muscle fibres (Vinsant et al., 2013) are already denervated. This denervation was not seen in the slow‐twitch muscle *soleus* and preceded changes in gait and grip strength (Vinsant et al., 2013). Mitochondria are localised to the NMJ both pre‐ and post‐synaptically due to the high energy demands of neurotransmission. At this same time point of denervation, mitochondria were fewer in number but larger in size in SOD1^G93A^ mice compared to controls, and the number of docked synaptic vesicles was reduced in both the TA and *soleus*, without a change in total synaptic vesicle number (Gould et al., 2006; Vinsant et al., 2013). Mitochondrial dysfunction is an early feature of SOD1^G93A^ mice, and the reduced number of mitochondria at the NMJ is a further indication of impaired axonal transport (Bilsland et al., 2010). Changes were also observed at the post‐synaptic region, including shorter post‐synaptic folds (Vinsant et al., 2013) and accumulation of SOD1 aggregates in the muscle (Turner et al., 2003). Functional examination of the diaphragm using electrophysiology in SOD1^G93A^ mice at P41 showed enhanced EPP amplitudes and quantal content, which could be interpreted as a compensatory mechanism for ongoing denervation (Rocha et al., 2013), and a decline in motor unit number and whole muscle force of fast‐twitch muscles (TA, GC, *extensor digitorum longus* [EDL]) (Hegedus et al., 2007). It therefore seems that morphological changes at the NMJ both pre‐ and post‐synaptically are a very early event in the SOD1^G93A^ mouse, leading to abnormal muscle function, particularly in fast‐twitch muscles.

While there is no loss of motor neuron cell body number or axon number at this pre‐symptomatic stage, there is evidence of pathology within the motor neuron and the ventral horn of the spinal cord. At P30–47, there are reports of astrogliosis, the formation of cytoplasmic vacuoles from mitochondria, endoplasmic reticulum or Golgi apparatus, mitochondrial changes such as swollen, mega‐mitochondria or vacuolated mitochondria and functional abnormalities including reduced generation of ATP (Chiu et al., 1995; Fischer et al., 2004; Gould et al., 2006; Vinsant et al., 2013).

It is not until early symptomatic stages that slow‐twitch muscles begin to show signs of pathology. For example, the *soleus* showed denervation and muscle atrophy at around P70, and a drop in motor unit number and muscle force was not detected until P90 (Narai et al., 2009). Other changes at this symptomatic stage include hindlimb loss of post‐synaptic structural proteins such as nestin, dystrophin, LRP4 and rapsyn, which maintain NMJ integrity, whereas in the forelimb muscles, only LRP4 was significantly reduced with a slower rate of decline compared to the hindlimb (Clark et al., 2016).

There is some reported variation in this timeline. Some studies did not detect any NMJ changes or clinical impairment until later ages, with denervation at P47 in GC, *soleus* and TA, and motor dysfunction at P75 (Fischer et al., 2004), or denervation at even later ages: at P60 in *cutaneous maximus* muscle (Tallon et al., 2016); at P81 in diaphragm, neck and hindlimb muscles (Schaefer et al., 2005); or P84 in GC and forelimb extensor muscles (Clark et al., 2016). Despite this delayed evidence of NMJ denervation, the pathology still preceded motor deficits and may indicate variation in strains (i.e., genetic drift), or the impact of local environmental conditions across mouse models housed in different research institutes.

P90–120 is considered the symptomatic stage of SOD1^G93A^ mice due to the decline in motor function. With the dramatic increase in denervated NMJs and onset of motor neuron death, clinical impairment becomes more apparent, with slowed weight gain, tremor and muscle weakness (Chiu et al., 1995; Fischer et al., 2004; Gurney et al., 1994; Schaefer et al., 2005). Some NMJs show transmission failure, while others show signs of compensation with sprouts, poly‐innervation, enhanced EPP amplitude and quantal content (Rocha et al., 2013; Schaefer et al., 2005). At the terminal stage of SOD1^G93A^ mice (P120–150), agrin, which induces AChR clustering, is overexpressed at the NMJ and around the muscle fibres in *soleus*, but with no change in AChR clusters (Narai et al., 2009). At around P140 pathological changes start to appear in the NMJs of forelimb muscles (Clark et al., 2016), followed by complete muscle paralysis and premature death (Gurney et al., 1994; Vinsant et al., 2013).

Although variations in the precise timing of NMJ pathology were reported by different groups, NMJ dysfunction consistently preceded motor dysfunction and motor neuron death in the SOD1^G93A^ mice in all studies. Potential explanations for variability in this model include the investigator's choice of time point and experimental design, the sensitivity of behavioural tests chosen and intrinsic differences in SOD1^G93A^ mice across institutes such as their genetic background and sex. In fact, a meta‐analysis reported that SOD1^G93A^ mice bred on the C57BL/6 background show delayed disease onset and a longer lifespan compared to those bred on the B6/SJL background (Pfohl et al., 2015). An effect on sex was only significant for mice on the B6/SJL background, where females survived longer than males (Pfohl et al., 2015).

### 2.2 NMJ changes in the SOD1^G37R^ mouse model

Less common are studies using the SOD1^G37R^ mouse model, which shows motor dysfunction and motor neuron cell death from around 300 days, with a median lifespan of 400–500 days (Ezzi et al., 2010). There is high variability in the phenotypes of this model, including its NMJ pathology, likely due to differing mutant protein expression levels and genetic backgrounds (Wong et al., 1995). In addition, Martineau et al. reported sex‐specific differences in this ALS mouse model. In vivo imaging of motor units and their NMJs in the TA muscle revealed more sprouts occurring over a 10‐week period in female mice than in males (Martineau, Di Polo, et al., 2020). This increase in motor unit size was surprisingly associated with an earlier decline in motor function in female SOD1^G37R^ mice, assessed by all‐limb grip strength, and more motor neuron degeneration in the lumbar spinal cord, suggesting that excessive motor unit expansion might lead to increased energy demands and therefore an accelerate disease progression (Martineau, Di Polo, et al., 2020).

In the pre‐symptomatic stages of SOD1^G37R^ mice, there is evidence of pathology in the motor neurons and altered electrophysiological readouts. Interestingly, changes in synaptic transmission precede morphological changes at the NMJ. Vacuolations in the ventral horn and proximal axons of motor neurons in all spinal cord levels were reported as early as P77 (Wong et al., 1995). At P180, while the NMJ structure remained intact, EPP and quantal content were increased in slow motor units and decreased in fast‐fatigable motor units (Tremblay et al., 2017). Disease onset and rate of progression in SOD1^G37R^ mice also vary greatly between studies. Between P120 and P330, these mice develop ‘clinical’ phenotypes of motor weakness, correlating with motor neuron cell death. SOD1^G37R^ mice showed an early decrease in spontaneous movement and muscle weakness at P120–180, with a significant reduction in motor neuron numbers by P140 (Wong et al., 1995). There is further evidence of a decrease in muscle strength at P294, and muscle paralysis with loss of motor neurons by P329 (Kriz et al., 2002). At this stage, it seems that the NMJ is undergoing a slow degeneration process. Martineau et al. used repeated in vivo imaging of the NMJ in TA to assess individual NMJs over time. They report a slow degeneration of the NMJ over a month before axonal degeneration occurs (Martineau et al., 2018). The slow rate of progression of this phenotype allows those motor neurons that are not yet fully degenerated to sprout and reinnervate (Martineau et al., 2018).

At the late symptomatic stage, Tremblay et al. reported a drop of 10% of body weight, with significant denervation of the fast‐fatigable motor units in EDL and signs of reinnervation such as poly‐innervation and sprouting in the slow and fast‐fatigue resistant motor units in *soleus*. By P450, all evidence of structural pathological changes at the NMJ had increased, including denervation but also signs of re‐innervation (Tremblay et al., 2017).

### 2.3 Alterations of terminal Schwann Cells (tSCs) in SOD1 mice

Multiple studies have reported structural and functional abnormalities of tSCs in the SOD1^G93A^ mouse model. While some found no change in tSC number at end stage in *diaphragm*, neck, EDL and *soleus* (Schaefer et al., 2005), others reported that prior to denervation in GC and *soleus*, some NMJs were not associated with a tSC cell body and were instead covered by processes which extended from pre‐terminal Schwann cells (Carrasco, Seburn, et al., 2016). Other NMJs had tSC cell bodies that were abnormally located outside of the perimeter of AChR labelling (Carrasco, Seburn, et al., 2016). With disease progression, denervated NMJs showed a complete loss of S100 and P75^NTR^ labelling (Carrasco, Seburn, et al., 2016; Harrison & Rafuse, 2020; Liu et al., 2013). The selective loss of tSCs from type IIb muscle fibres may be due to tSC overexpression of Sema3A, a neuronal regeneration inhibitor, making them vulnerable to phagocytosis by infiltrating macrophages (Harrison & Rafuse, 2020). Interestingly, S100, but not P75^NTR^, labelling was not detected in the extraocular muscles of SOD1^G93A^ mice, despite the maintenance of NMJs even at end stage (Liu et al., 2013).

The absence of tSC cell bodies has also been seen prior to denervation in other SOD1 models, such as the SOD1^G85R^ mice in GC (Carrasco, Seburn, et al., 2016) and following induced denervation in GC, *soleus* and *plantaris* in asymptomatic SOD1^G93A^ and SOD1^G85R^ mice (Carrasco, Bahr, et al., 2016; Harrison & Rafuse, 2020). Characterising the molecular profile of tSCs in general, and across the different fibre types specifically, will help our understanding of their role in denervation and re‐innervation in ALS. For example, there is evidence of changes in the electrophysiological properties of tSCs in *soleus* and *sternomastoid* in pre‐symptomatic SOD1^G37R^ mice (Arbour et al., 2015; Martineau, Arbour, et al., 2020). This implies a functional impairment of tSCs in SOD1 mouse models, which could affect their phagocytic activity and repair capacity during nerve degeneration.

## 3 C9ORF72

The most common gene mutation in sporadic and familial ALS from European populations is a GGGGCC (G_4_C_2_) hexanucleotide repeat expansion (HRE) in the first intron and non‐coding region of the C9orf72 gene. Healthy subjects usually have up to 23 copies of the G_4_C_2_ repeat, whereas ALS patients can carry hundreds to thousands of repeats (DeJesus‐Hernandez et al., 2011; Renton et al., 2011). This massive expansion of the intronic G_4_C_2_‐repeat leads to pathological toxic gain of function and loss of function mechanisms in motor neurons, affecting processes including nucleocytoplasmic transport, RNA processing and autophagy (Lai & Ichida, 2019). Despite C9orf72's involvement in many major cellular regulatory processes, direct involvement at the peripheral nervous system, with pathologies such as denervation and motor deficits, has not yet been shown consistently across models. The logistics of inserting a transgene containing such a large repeat expansion has hampered the development of suitable models. Here, we aim to shed light on the involvement of the peripheral nervous system in the C9orf72 phenotype across various ALS models.

### 3.1 NMJ changes in C9orf72 BAC mouse models

Mouse models to date have largely utilised bacterial artificial chromosome (BAC) constructs or adeno‐associated viral vectors to overexpress human C9orf72 with G_4_C_2_ repeat expansions of variable length. Mouse lines termed C9‐BAC showed a sex‐dependent disease phenotype which correlated severity with repeat length and expression (Liu et al., 2016). C9‐37 with a single copy of 37 short repeats caused no disease phenotype, whereas even short repeats with increased copies of the BAC (C9‐36/29; i.e., high expression of short repeats) triggered mild disease progression. C9‐500/32 and C9‐500, with transgenes expressing copies containing a mix of ~500 and 32 repeats or ~500 repeats alone, showed a more rapid and severe disease progression (Liu et al., 2016). Up to 35% of female mice from the C9‐500/32 and C9‐500 lines developed sudden weight loss and paralysis that led to death at 20–40 weeks of age, accompanied by significant NMJ denervation of *tibialis anterior* and diaphragm muscles, loss of motor neurons and muscle atrophy. Males suffering from acute disease progression died past the age of 40 weeks and mice, including females, from lines expressing a reduced copy number of G_4_C_2_ (C9‐37 line) were significantly healthier than lines with increased copy numbers (Liu et al., 2016). While Liu et al. reported a significant motor phenotype by the age of 5–7 months, or less severe pathology in slow progressive lines with fewer repeat expansions by 12–14 months, O'Rourke et al. observed no motor phenotype even at old age (O'Rourke et al., 2015). Despite these mice carrying a BAC full‐human C9orf72 expansion, no denervation or structural changes of the NMJ were noted in TA, even though sense and antisense RNA foci accumulated throughout the nervous system as early as 3 months of age (O'Rourke et al., 2015). Similar pathology of accumulation of RNA foci at 3 months of age and lack of motor and behavioural phenotypes until old age was also observed in a mouse model carrying a BAC human C9orf72 gene containing exons 1–6 (Peters et al., 2015).

RNA‐foci and dipeptide repeat proteins (DPRs) are considered a pathological hallmark of C9orf72‐linked ALS/FTD. Herranz‐Martin et al. managed to shine some light on the relationship between RNA‐foci, DPRs and drivers of disease pathogenesis. AAV overexpression vectors delivered at P1 with 10 HREs or 102 interrupted repeats demonstrated that only mice expressing longer repeats (HRE‐102) developed RNA foci, which were observed in both models at 12 months of age (Herranz‐Martin et al., 2017). Only HRE‐102 mice demonstrated NMJ pathology in *lumbrical* muscles (abnormal pre‐synaptic neurofilament accumulation, nerve terminal blebbing, reduction in acetylcholine receptor density), behavioural impairment as well as memory deficits and gait impairment. Another model links C9orf72 to motor deficits. Doxycycline‐induced ubiquitous expression of C9orf72 with a 36 HRE under the hnRNP promoter resulted in 45% of mice developing a rapid onset of disease with reduced activity and loss of body weight leading to death within 2–3 weeks (Riemslagh et al., 2021). After 4 weeks of induced expression, mice showed significant muscle atrophy, denervation of NMJs in EDL and alteration in post‐synaptic endplate morphology, however, no change in the number of motor neurons was observed. Specific neuronal expression of the C9orf72 construct showed no RNA foci after 24 weeks of doxycycline administration (Riemslagh et al., 2021).

Most models assess insertion of HREs, as neuronal knockout of C9orf72 has been shown to not affect neuromuscular pathology (Koppers et al., 2015). Smcr8 forms a protein complex with C9orf72 and both play a role in autophagy regulation. Therefore, Liang et al. (2019) assessed dependence of Smcr9 on C9orf72 in mice deficient for Smcr8 and/or C9orf72. While mice lacking C9orf72 showed no neuromuscular pathology, as previously reported, mice lacking Smcr8 developed axonal swellings at the NMJ in the diaphragm, likely neurofilament accumulation, which worsened in double knockout mice as well as in C9‐BAC mouse models. Therefore, Smcr8's crucial function in autophagy regulation can mostly compensate for the loss of C9orf72, but C9orf72 cannot fully compensate for the loss of Smcr8, leading to impaired axonal transport (Liang et al., 2019).

**FIGURE 3:**
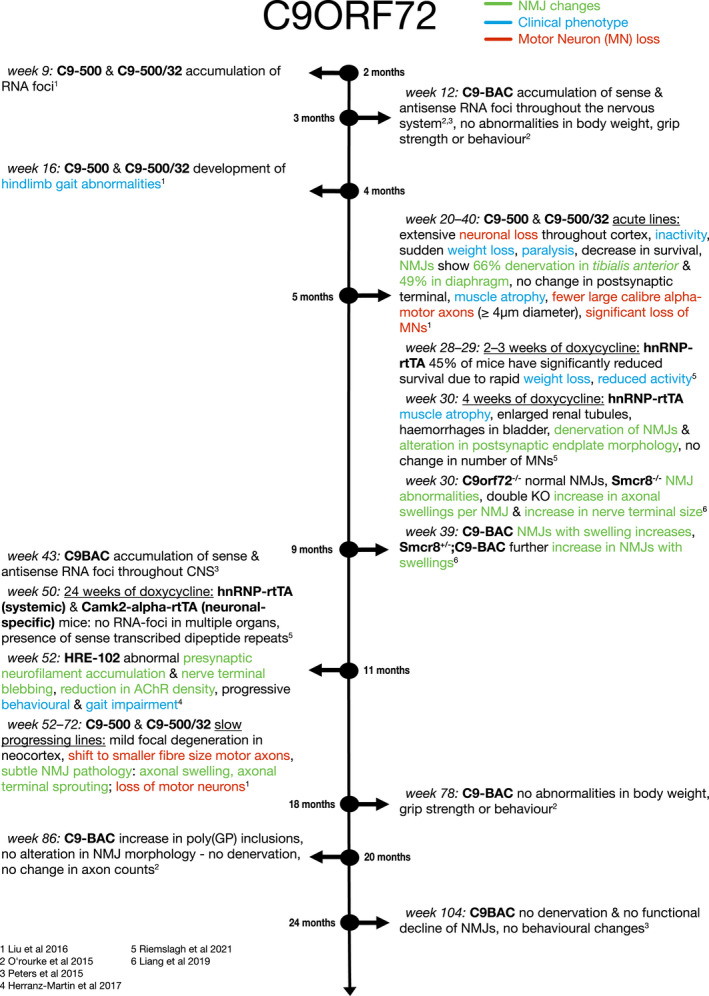
Timeline of NMJ denervation in C9orf72 mouse models. Blue descriptions indicate ‘clinical’ phenotypes. Green descriptions indicate NMJ changes. Red descriptions indicate motor neuron (MN) loss

Variation in length and expression levels of C9orf72 G_4_C_2_ repeats play a significant role in disease manifestation. Since most models do not show pathology at the NMJ or motor neuron cell death, it is difficult to determine the relationship between C9orf72‐specific molecular pathology and ALS. We still do not know the minimum repeat length and expression level required to cause neuromuscular pathology. However, all models that developed a motor phenotype showed signs of motor neuron degeneration and alterations of the pre‐synaptic NMJ, accompanied with signs of atrophy, as summarised in Figure 3 (Herranz‐Martin et al., 2017; Liu et al., 2016; Riemslagh et al., 2021). More research is needed to determine the order of progression of these phenotypes.

## 4 TDP‐43

TAR DNA‐binding protein‐43 (TDP‐43) is a highly conserved DNA/RNA‐binding protein that predominantly localises to the nucleus and acts as a transcriptional regulator (Ou et al., 1995), with roles across RNA metabolism (Prasad et al., 2019). The majority of ALS patients, regardless of genetic status, show cytoplasmic TDP‐43 positive protein aggregates in motor neurons, implicating this protein in ALS pathology (Neumann et al., 2006), while rare mutations in its gene, *TARDBP*, are found in some patients (Zou et al., 2017). Recent studies have shown that TDP‐43 is involved in axon outgrowth and is actively transported across motor neurons, therefore playing an important role in the overall regulation of axonal plasticity (Fallini et al., 2012; Hörnberg & Holt, 2013). Furthermore, TDP‐43 has been shown to localise to the pre‐synaptic nerve terminal at the NMJ, opposing post‐synaptic AChRs in healthy mice (Narayanan et al., 2013). In ALS, the pathological redistribution of TDP‐43 occurs along dendrites and axons, which leads to the hypothesis that dysregulated translation along axons plays a major role in the degeneration of motor neurons (Fallini et al., 2012). However, it is still unclear whether a gain of function through toxic accumulation of aggregates, a loss of function in the nucleus or both drives disease pathogenesis.

TDP‐43 has proven to be essential for prenatal development and survival, since knockout of TDP‐43 is embryonically lethal (Kraemer et al., 2010). Many overexpression models of TDP‐43 show early disease onset and rapid disease progression as well as pathologies not present in human patients such as gastrointestinal dysfunction (Esmaeili et al., 2013; Guo et al., 2012). Here, we describe the few TDP‐43 models that have shown involvement of the peripheral nervous system, with pathology such as structural NMJ abnormalities, and defects in axonal branching and synapse formation.

### 4.1 NMJ changes in the TDP‐43^Q331K^ mouse

There is a considerable variation in onset of pathology across mouse models harbouring TDP‐43 mutations or expressing wild‐type TDP‐43. This is best illustrated by comparing mouse models expressing human TDP‐43^Q331K^. Despite breeding on the same C57B6/J genetic background, there are differences in time of onset, sex difference and TDP‐43 dose‐dependent motor neuron phenotype across studies (Arnold et al., 2013; Mitchell et al., 2015; Shan et al., 2010; White et al., 2018).

A mouse model with neuron‐specific, postnatally expressed human TDP‐43 in C57BL/6;SJL mice correlated in phenotype severity with the copy number of transgenes among mouse lines (Shan et al., 2010). The most severe, highest TDP‐43 copy number mouse line died at 3 weeks of age. Male mice were particularly susceptible to weight loss and motor phenotypes from 2 weeks of age, in comparison to female mice that only developed a fine tremor at 3 months of age. Shan et al. demonstrated further that normal TDP‐43 levels are required for neuromuscular maintenance. MitoCFP;TDP‐43 compound mice, expressing fluorescently labelled mitochondria in neurons, revealed fewer mitochondria at the NMJ in TA, indicative of disruption to mitochondrial transport (Wang et al., 2013), as well as plaque‐like NMJs, which are characteristic of immature NMJs in neuromuscular dysfunction and/or developmental delay. Abnormal NMJs were accompanied by muscle weakness and 20% reduction in muscle fibre size in 3‐week‐old TDP‐43 mice (Shan et al., 2010).

To assess the effects of mutant TDP‐43 on NMJs and motor phenotypes, Arnold et al. (2013) investigated TDP‐43^Q331K^, TDP‐43^Q331K‐Low^ and TDP‐43^M337V^ mutant overexpression models compared to TDP‐43^WT^, overexpressing wild‐type human TDP‐43. At 3 months of age, TDP‐43^Q331K^ mice demonstrated an onset of tremor, hindlimb clasping and reduction in hindlimb grip strength, which progressively increased with age until 10 months, where there was no further loss of motor function. At 10–12 months, the TDP‐43^Q331K^ model showed a reduction in total number of motor axons, primarily a loss of large alpha‐motor axons (>3.5 µm diameter), a 30% reduction in number of NMJs in *gastrocnemius*, accompanied with muscle atrophy and centralised nuclei in the GC, which suggests muscle remodelling (Arnold et al., 2013). TDP‐43^Q331K‐Low^ and TDP‐43^M337V^ mutants showed no difference in neuromuscular pathology in comparison to TDP‐43^WT^. These models suggest a period of active neurodegeneration until the age of 10 months, without a significant increase in pathology thereafter. Furthermore, Arnold et al. showed that degeneration of lower motor neurons can occur without loss of TDP‐43 from the nucleus and without accumulation of insoluble TDP‐43, the latter being considered a hallmark of ALS (Arnold et al., 2013). Despite assessing the same TDP‐43^Q331K^ mutant model, Chand et al. observed no motor deficits at the age of 3 months old. However, alterations at the NMJ, such as functional decline characterised by an increase in MEPP amplitude and decreases in MEPP frequency in EDL, as well as increased poly‐innervation at the NMJ in TA, were observed (Chand et al., 2018). Defective vesicle cycling was suggested to play a role in the further functional decline at the NMJ at 10 months of age, as seen by a progressive decrease in synaptic vesicle to acetylcholine receptor volume ratio and acetylcholine receptor volume. Furthermore, at 10 months of age, mice had a significant loss of lumbar motor neurons and developed a motor phenotype (Chand et al., 2018).

Mitchell et al. assessed the same TDP‐43^Q331K^ mutant model as generated by Arnold et al. in the same housing conditions. However, they compared it to heterozygous TDP‐43^WTxQ331K^ mutants that co‐express human WT and human mutant TDP‐43. As opposed to the non‐fatal model of TDP‐43^Q331K^, TDP‐43^WTxQ331K^ mutants suffered a 25% loss in body weight by the age of 8–10 weeks where they reached the humane endpoint (Mitchell et al., 2015). This model shows a more accelerated neuromuscular phenotype, with onset of tremor followed by a rapid decline in motor function at the age of 3 weeks, as opposed to the slower onset of motor impairment in TDP‐43^Q331K^ mutants at 3 months of age. Heterozygous mutants displayed progressive severe hindlimb paralysis and abnormal hindlimb splay at the age of 5–6 weeks, followed by ~46% loss of large calibre alpha‐motor axons and reduction in overall AChR area at 8 weeks. Assessment of NMJ morphology at the age of 24 months in TDP‐43^Q331K^ mutants showed a reduction in AChR area in GC and ~35% less intact NMJs compared to age‐matched controls (Mitchell et al., 2015).

CRISPR‐Cas9 has also been used to induce the equivalent TDP‐43^Q331K^ point mutation in the endogenous mouse *Tardbp* gene (White et al., 2018). Comparison of motor phenotype, TDP‐43 pathology and cognitive behaviour of TDP‐43^Q331K/Q331K^ and TDP‐43^Q331K/+^ mutants suggested not only that males are more susceptible to deleterious effects of TDP‐43^Q331K^ but also demonstrated potential compensatory transcription of genes in lumbar motor neurons that might exhibit a neuroprotective effect. Normal morphology and number of motor neurons lacking TDP‐43 aggregates, as well as fully innervated NMJs in GC at 5 months were associated with upregulation of transcripts that play a role in NMJ plasticity (White et al., 2018). RNA‐Seq showed upregulation of agrin in motor neurons, which acts as a synaptic organiser of the NMJ during development and maturation of the NMJ. The crucial role of agrin in neuromuscular diseases has previously been shown in mouse models of spinal muscular atrophy (SMA), where agrin mRNA was shown to be mis‐spliced at pre‐symptomatic time points prior to motor neuron involvement, as well as other genes involved in developing motor circuitry (Zhang et al., 2013). A lack of agrin in these SMA models has been associated with NMJ pathology in diaphragm, and subsequent treatment with agrin rescued motor impairment and delayed disease progression (Boido et al., 2018). Therefore, upregulation of agrin has been suggested to promote NMJ function and plays a role in the resilience of NMJs to TDP‐43^Q331K^ mutation in this mouse model. As opposed to TDP‐43^WTxQ331K^ mutants (Mitchell et al., 2015), both TDP‐43^Q331K/Q331K^ and TDP‐43^Q331K/+^ mutants lacked any motor impairment in comparison to weight‐matched wild‐type mice (White et al., 2018). Furthermore, NMJs in the GC showed normal morphology as well as physiology and were fully innervated at the age of 18–23 months. Similarly, mouse models lacking TDP‐43 in oligodendrocytes have a more aggressive neurological phenotype such as tremor and changes in gate, yet fully lack neuromuscular denervation (Wang et al., 2018).

### 4.2 NMJ changes in the TDP‐43^M337V^ and TDP‐43^G298S^ mouse

Some groups have focussed on other ALS‐associated *TARDBP* mutations, such as M337V and G289S. The M337V mutation was found to be relatively common in a small group of patients with *TARDBP* mutations, bearing in mind that these mutations as a whole are quite rare, and in this group, the G298S mutation was found to correlate with the most severe disease progression (Corcia et al., 2018). Ebstein et al. created knock‐in models of both TDP‐43^M337V^ and TDP‐43^G298S^ by introducing the human mutation into the mouse *Tardbp* gene. Both of these mouse models produced a mild motor neuron degenerative phenotype, with no evidence of a change in motor neuron number until 2.5 years of age (Ebstein et al., 2019). Analysis of NMJs in the TA showed no signs of denervation until 1.5 years, where they found 3% denervated in the homozygous TDP‐43^G298S^ model, but no evidence of denervation in the TDP‐43^M337V^ model until 2.5 years of age where 5% of NMJs were denervated. This group also assessed the slow‐twitch *soleus* muscle and mixed fibre type GC muscle at 2.5 years, but only 1.5% NMJs were denervated in the *soleus* in both models (Ebstein et al., 2019). Since there was no evidence of motor neuron loss at any time point, these models may indicate that denervation precedes motor neuron cell body loss, but since levels of denervation were minimal, it is difficult to draw certain conclusions. Another M337 knock‐in model introduced the whole human *TARDBP* gene including intronic and regulatory elements at the *Rosa26* locus (Gordon et al., 2019). This model appears to be slightly more progressive than the Ebstein et al. model, demonstrating motor dysfunction and reduced grip strength at 9 months. At this time point, there was evidence of NMJ degeneration in the form of decreased post‐synaptic area in the *lumbrical* muscles, but no changes in GC NMJs (Gordon et al., 2019).

### 4.3 NMJ changes in systemic versus neuron‐specific TDP‐43 models

Expressing TDP‐43^A315T^ under the pan‐neuronal Prp promoter, the mouse mutant Prp‐TDP‐43^A315T^ produced notable neuromuscular pathology (Coyne et al., 2017). TDP‐43 was shown to interact with Hsc70‐4, a chaperone protein normally expressed in high levels at the NMJ and known to facilitate synaptic vesicle cycling. Overexpression of TDP‐43^A315T^ led to a reduction in synaptic Hsc70‐4 by 21% in mice at 12 weeks of age (Coyne et al., 2017). Mutant TDP‐43 caused inhibition of Hsc70‐4 mRNA translation, therefore reducing Hsc70‐4 protein levels at the NMJ, leading to impaired synaptic vesicle endocytosis (Coyne et al., 2017).

TDP‐43^A315T^ pathology has been shown to be much less severe than other TDP‐43 mutants, such as TDP‐43^G348C^ or TDP‐43^N390D/+^ (Huang et al., 2020; Swarup et al., 2011). Assessment of ubiquitous, rather than neuron‐specific, expression of TDP‐43^A315T^ and TDP‐43^N390D^ in homozygous and heterozygous mutants supports the male‐dominant, TDP‐43 dose‐ and age‐dependent pathology. TDP‐43^A315T^ mutants express lower levels of TDP‐43 than homozygous TDP‐43^N390D^ mutants, and thus present a more severe phenotype with onset of significant motor dysfunction at 6 months and denervation of NMJs in *soleus* (Huang et al., 2020).

Human wild‐type TDP‐43, TDP‐43^A315T^ or TDP‐43^G348C^ mutants, respectively, demonstrate increasing severity in cognitive impairments as well as motor phenotype (Swarup et al., 2011). At 42 weeks, age‐related motor deficits are accompanied by denervation of NMJs and a decrease of large calibre motor axons (6–9 µm), most prominent in the TDP‐43^G348C^ mutant. Furthermore, cytoplasmic inclusions of TDP‐43, considered a hallmark of ALS, are more severe in the TDP‐43^G348C^ mutant, also suggesting a TDP‐43 dose dependency of pathology (Swarup et al., 2011).

The argument that global expression of TDP‐43 is required, and that disease‐mechanisms strongly depend on mutant TDP‐43 interacting with multiple cell types and not only neuronal tissue, has been demonstrated by Iguchi et al. and Ditsworth et al. In comparing their previously described TDP‐43^Q331K^ mutant model to TDP‐43^Q331K/VChAT‐Cre^ mice, with reduced mutant TDP‐43 expression in motor neurons via Cre‐recombinase‐mediated excision of TDP‐43^Q331K^, they show that despite a significant delay in onset of motor symptoms and neuromuscular degeneration, age‐dependent degeneration is not prevented (Arnold et al., 2013; Ditsworth et al., 2017). Comparison of these models shows that motor performance is unaffected at 10 months of age in TDP‐43^Q331K/VChAT‐Cre^ mice. Additionally, motor neuron cell bodies show no degeneration at the age of 18 months in comparison to TDP‐43^Q331K^ mutants (Ditsworth et al., 2017). However, reduced TDP‐43 expression specific to motor neurons only attenuated disease onset. TDP‐43^Q331K/VChAT‐Cre^ mice suffered alterations at the NMJ in GC later in disease progression, such as fragmentation or reduction in endplate size, from 19 months onwards (Ditsworth et al., 2017). Whereas Ditsworth et al. show a more moderate phenotype, Iguchi et al. (2013) demonstrate a more severe motor phenotype upon motor neuron‐specific TDP‐43 knockout (TDP CKO mice). At 11 months of age, TDP CKO mice develop a tremor and progressive denervation of NMJs in GC, leading to progressive weight loss and muscle atrophy of trunk and hindlimb at 13–14 months of age (Iguchi et al., 2013). This highlights in particular that focus on the preservation of the peripheral nervous system alone via reduction of targeted TDP‐43 expression might be an ineffective strategy to treat ALS (Ditsworth et al., 2017).

### 4.4 NMJ changes in hTDP‐43^∆NLS^ mice

In order to force a mislocalisation of TDP‐43 out of the nucleus and into the cytoplasm, a mouse model expressing human TDP‐43 without its nuclear localisation signal (hTDP‐43^∆NLS^) was created, with ∆NLS transcription that was suppressible via use of doxycycline (Walker et al., 2015). After 5 weeks of age, expression of hTDP‐43^∆NLS^ was induced by removal of doxycycline from the animals' food, which led to rapid onset of disease. Two weeks after induction of hTDP‐43^∆NLS^ expression, there was onset of motor symptoms and early cytoplasmic accumulation of hTDP‐43 was visible. Two weeks later (4 weeks of hTDP‐43^∆NLS^ expression), mice suffered from brain atrophy and significant denervation of NMJs in TA, without change in motor neuron numbers, suggesting a dying‐back pathology (Walker et al., 2015). Mice progressively lost ~28% of lower spinal cord motor neurons and ~60% of NMJs were denervated after 6 weeks of hTDP‐43^∆NLS^ expression. This was followed by an ~50% decrease in motor neurons, grouped fibre atrophy and centralised nuclei after 8‐week hTDP‐43^∆NLS^ expression (Walker et al., 2015). Grouped fibre atrophy and centralised nuclei are a strong indicator of prolonged denervation and reinnervation processes (Roman & Gomes, 2018). Neuromuscular pathology of hTDP‐43^∆NLS^ mice led to their death after 8 weeks of hTDP‐43^∆NLS^ expression. However, subsequent suppression of hTDP‐43^∆NLS^ expression via doxycycline led to muscle re‐innervation and functional recovery of the mouse model, as nuclear TDP‐43 increased and progressive motor neuron loss was prevented (Walker et al., 2015). This provides hope that while NMJ dysfunction is an early feature of TDP‐linked ALS pathology, it may well be reversible.

**FIGURE 4:**
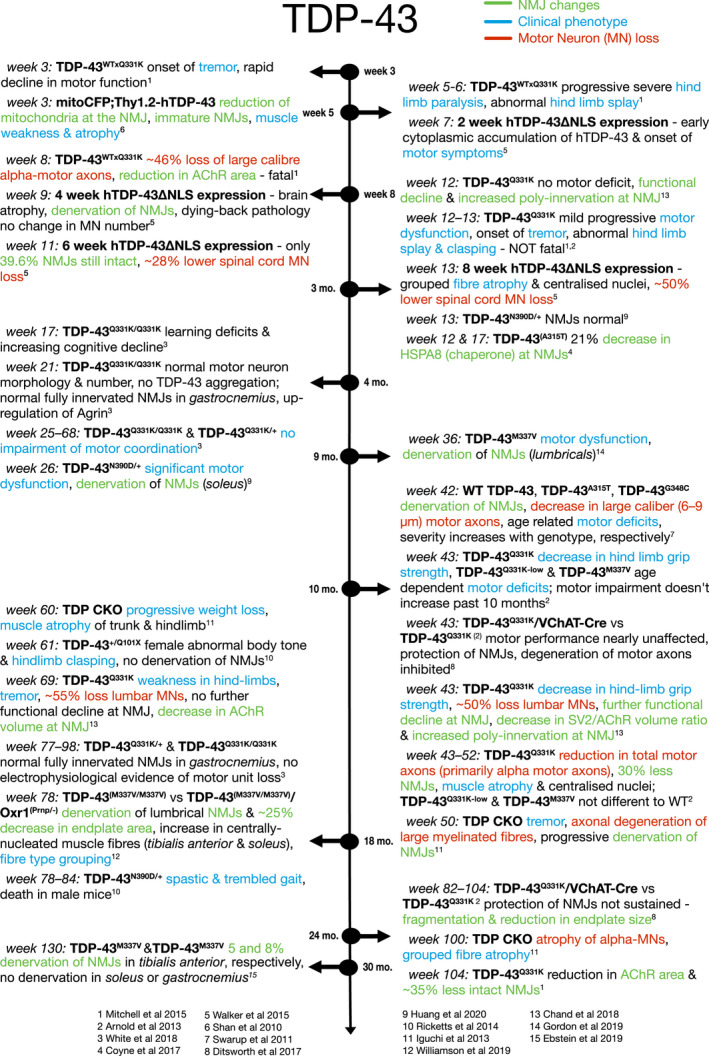
Timeline of NMJ denervation in TDP‐43 mouse models. Blue descriptions indicate ‘clinical’ phenotypes. Green descriptions indicate NMJ changes. Red descriptions indicate motor neuron (MN) loss

The variety of mouse models detailed above has brought a plethora of knowledge regarding TDP‐43 function and potential therapeutic avenues that can be explored, such as utilisation of oxidation resistance 1 protein (Oxr1) to ameliorate the neuromuscular phenotype of ALS (Williamson et al., 2019). However, the variable severity that can be observed is not only promoter driven and dependent on knock‐in or knock‐out of TDP‐43 expression. The same genetic manipulation on the same background has shown different severity across mouse lines, which recapitulates the heterogeneity observed in humans (Shan et al., 2010). Overall, the majority of TDP‐43 models that develop a significant motor phenotype also demonstrate morphological alterations of the NMJ as summarised in Figure 4. These alterations seem to be strongly TDP‐43 mutation‐dependent, and therefore also dose‐ and age‐dependent.

## 5 FUS

FUS (fused in sarcoma, also known as TLS) mutations are found in 4–6% of familial ALS patients and less than 1% of sporadic ALS patients, depending on the population (Zou et al., 2017). There are more than 50 known ALS‐linked mutations in FUS, which are mostly located in the nuclear localisation signal (Deng et al., 2014) leading to cytoplasmic mislocalisation and formation of aggregates (Hewitt et al., 2010; Neumann et al., 2009). Under physiological conditions, FUS is localised to the nucleus and functions as a DNA/RNA‐binding protein, with diverse roles including splicing, mRNA transport and microRNA processing (Lagier‐Tourenne et al., 2012). As such, FUS and TDP‐43 have overlapping, as well as distinct, roles within the neuron (Kapeli et al., 2016). Using super resolution microscopy, FUS was shown to localise to the pre‐synaptic terminal of the NMJ (So et al., 2018).

### 5.1 NMJ changes in FUS knockdown models

**FIGURE 5:**
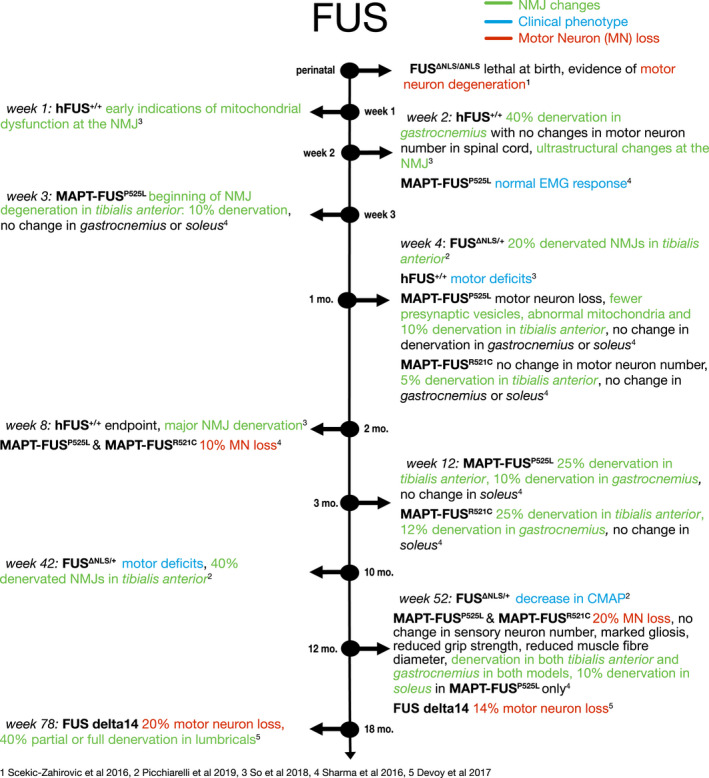
Timeline of NMJ denervation in FUS mouse models. Blue descriptions indicate ‘clinical’ phenotypes. Green descriptions indicate NMJ changes. Red descriptions indicate motor neuron (MN) loss

There is debate over whether FUS‐linked ALS pathology is caused by a loss of nuclear function due to cytoplasmic mislocalisation, or is due to a toxic gain of function, perhaps in part due to the formation of FUS‐positive aggregates. As such, modelling FUS‐linked ALS can either be through a knock‐down of endogenous FUS or a knock‐in of mutant FUS (Figure 5). Complete knockout of FUS is postnatally lethal, with newborn pups dying from respiratory difficulties (Scekic‐Zahirovic et al., 2016). Removing the nuclear localisation signal from FUS (FUS^ΔNLS^) leads to mislocalisation of FUS to the cytoplasm, and mice homozygous for this deletion (FUS^ΔNLS/ΔNLS^) died postnatally in a similar fashion to FUS knockout mice, with evidence of motor neuron degeneration (Scekic‐Zahirovic et al., 2016). FUS^ΔNLS/ΔNLS^ mice showed reduced endplate areas and fewer endplates in the TA and GC, which did not correlate with a change in the number of muscle fibres (Picchiarelli et al., 2019). Electron microscopy revealed altered morphology in both pre‐ and post‐synaptic compartments of the NMJ and a loss of secondary post‐synaptic invaginations. This indicated that the pathology had a post‐synaptic origin, and restoring nuclear FUS expression selectively in the muscle rescued the changes in endplate area, although did not change the postnatal lethality (Picchiarelli et al., 2019).

To avoid the issue of early postnatal lethality, heterozygous FUS^ΔNLS/+^ mice were assessed. FUS^ΔNLS/+^ mice show motor deficits beginning at around 10 months, which is preceded by NMJ pathology. Denervation at the NMJ of TA muscle was apparent at 1 month old: around 20% of NMJs appeared vacant with a smaller endplate area (Picchiarelli et al., 2019). By 10 months old, 40% of NMJs were denervated in the TA. Heterozygous FUS^+/−^ mice showed no change in innervation or endplate area up to 28 months old, indicating that it is not simply the loss of nuclear FUS causing pathology but an additional gain‐of‐function pathology from cytoplasmic mislocalisation. Mechanistically, FUS was found to be enriched in subsynaptic nuclei of myofibres, where it can regulate expression of AChR subunits through interaction with their promoter (Picchiarelli et al., 2019).

### 5.2 NMJ changes in FUS knock‐in models

Further evidence that FUS pathology begins at the NMJ comes from mice overexpressing WT human FUS (hFUS). hFUS^+/+^ mice develop a progressive motor phenotype with a lifespan of around 56 days (So et al., 2018). These mice develop motor symptoms from 4 weeks of age (Mitchell et al., 2013). Pre‐symptomatically, at P15, hFUS^+/+^ mice show around 40% denervation in the *gastrocnemius* with no change in motor neuron number in the lumbar region of the spinal cord (So et al., 2018). At this time point, ultrastructural analysis of the NMJ showed a loss of synaptic vesicles, disrupted membranes and a decrease in the number of mitochondria at the nerve terminal, while the post‐synaptic endplate appeared relatively healthy compared to controls (So et al., 2018).

In order to determine differences in NMJ pathology across different muscle types, a mouse model overexpressing mutant FUS under the MAPT neuronal promoter was used (Sharma et al., 2016). Loss of motor neurons with the most severe mutation, FUS^P525L^, was evident from P30, with a delay in motor neuron loss with other mutations and WT FUS. Preceding this loss of motor neurons in the lumbar region of the spinal cord, NMJs in the TA, comprised of fast‐fatigable motor units, were around 10% denervated at P20 although there was no evidence of denervation in the GC or *soleus* (Sharma et al., 2016). NMJs in the GC, comprising a mix of slow and fast fatigable motor units, showed denervation from P90 onwards, whereas in the *soleus*, which is innervated almost entirely by slow motor units, there was only evidence of denervation at P360 (Sharma et al., 2016). There was no evidence of denervation at any time point in mice overexpressing wild‐type human FUS in neurons (Sharma et al., 2016). Since this model shows a neuron‐specific expression, this indicates a pre‐synaptic mechanism of pathology, and the evidence of NMJ denervation prior to motor neuron cell death shows a dying‐back pathology. Combined, these studies highlight that NMJ pathology is evident prior to motor neuron loss in the spinal cord, and that this may be due to loss of NMJ‐specific roles of FUS at the pre‐synaptic terminal and in the sub‐synaptic nuclei of the myofibre as well as toxic gain‐of‐function mechanisms impacting pre‐synaptic vesicles and mitochondria.

To produce a model that more closely mimicked FUS pathology in ALS patients, the FUS delta14 model was created where a frameshift mutation was knocked into the endogenous mouse *Fus* gene, as well as the human exon 15 coding sequence (Devoy et al., 2017). Since this gene was under the control of the endogenous *Fus* promoter, expression of the mutated protein was at physiological levels. These mice show a milder disease progression, with some motor neuron loss evident at 12 months old and around 40% fully or partially denervated NMJs of *lumbrical* muscles at 18 months old. While there was only a slight effect on survival in this FUS mouse model, genes associated with mitochondria and translational machinery were dysregulated, and there was cytoplasmic mislocalisation of FUS with no aggregation (Devoy et al., 2017).

## 6 THERAPEUTIC INTERVENTIONS TO MAINTAIN NMJ INNERVATION IN ALS

Several studies have proposed therapeutic strategies to support the health of the NMJ in ALS. For example, SOD1^G93A^ mice treated with an antibody blocking DR6 (death receptor 6; a tumour necrosis factor receptor) for 2 weeks before disease onset showed preserved NMJ integrity in GC muscle, decreased gliosis in spinal cord and improved motor function (Huang et al., 2013). Another strategy showed that Nogo‐A, an axon regeneration inhibitor, is overexpressed in muscles and correlates with disease severity (Jokic et al., 2005) and NMJ denervation (Bruneteau et al., 2015) in ALS patients. It is also upregulated in SOD1^G86R^ and SOD1^G93A^ mouse models (Bros‐Facer et al., 2014; Jokic et al., 2006), while SOD1^G86R^/Nogo‐A^−/−^ mice maintained their NMJ integrity, with an improved survival (Jokic et al., 2006). Weekly injections of an anti‐Nogo‐A antibody in symptomatic SOD1^G93A^ mice starting at P70 improved muscle force in the TA and EDL, as well as innervation of the EDL at P90 (Bros‐Facer et al., 2014). This approach was developed by Glaxo Smith Kline (GSK), which produced an anti‐Nogo‐A antibody Ozanezumab, although this failed to show any efficacy in clinical trials in comparison to a placebo (Meininger et al., 2017). Further work found that treatment with masitinib, a tyrosine kinase inhibitor, in partially denervated asymptomatic SOD^G93A^ mice decreased macrophage infiltration, which subsequently preserved tSCs associated with type IIb muscle fibres and improved reinnervation (Harrison & Rafuse, 2020). Excitingly, in a Phase II/III clinical trial, masitinib slowed disease progression and improved quality of life of patients when used as an add‐on therapy to riluzole, although there was no effect on overall survival (Mora et al., 2020). Similarly, muscle‐specific kinase (MuSK) overexpression in SOD1^G93A^ mice delayed onset and slowed the progression of NMJ denervation and motor dysfunction, but had no effect on lifespan (Pérez‐García & Burden, 2012). Another strategy could be the treatment with growth factors, for example, IGF‐1 which can preserve NMJ integrity following nerve transection (Apel et al., 2010) and was found to be beneficial in ALS models (Sakowski et al., 2009), although this failed in a Phase III clinical trial (Sorenson et al., 2008). While the majority of these approaches have so far failed to translate to human clinical trials, targeting NMJ integrity should continue to be considered as a therapeutic target due to its early role in disease progression, both in ALS patients and the majority of ALS models. Further investigation into the events surrounding NMJ degeneration across all ALS models, including other species such as larger animal models (separate review in this special edition), will highlight more therapeutic options and hopefully aid in better translation during human clinical trials in the future.

## 7 CONCLUSION

NMJ pathology is prevalent in ALS patient muscle samples, but the role that NMJ pathology plays in the progression of the disease is less well understood. Mouse models of ALS may be considered partly unreliable due to the heterogeneity of disease onset, and so findings from one mouse model may not necessarily translate to other models and indeed to human patients. Here, we have highlighted the differences in NMJ pathology across ALS models. The SOD1^G93A^ mouse model is the most studied due to its recapitulation of the human disease course with progressive muscle weakness and paralysis corresponding to loss of motor neurons, as well as the early discovery of *SOD1* mutations in ALS patients (Rosen et al., 1993). The SOD1^G93A^ mouse shows clear NMJ pathology prior to motor neuron loss, and indeed before motor symptoms appear, as summarised in Figure 2, implying that the NMJ is a primary site of pathology and supporting a dying‐back disease progression. However, the dying‐back hypothesis is less clear in the SOD1^G37R^ mouse model. Mouse models of TDP‐43, summarised in Figure 4, and FUS models summarised in Figure 5, also both show clear NMJ pathology prior to motor neuron cell death and again support the dying‐back hypothesis.

Conversely, mouse models expressing the most common gene mutation found in ALS patients, the C9orf72 repeat expansion, rarely show motor dysfunction or NMJ pathology. If they do, then there is not yet evidence of NMJ dysfunction prior to motor neuron loss, as summarised in Figure 3. In fact, the variability in these models makes it difficult to draw conclusions on C9orf72‐linked ALS disease progression at all. Some groups show a sex‐specific effect where female mice have a more severe phenotype (Liu et al., 2016). Other groups describe subsets of mice with the same genetic mutations that have an acute phenotype while other subsets with the same genotype show milder progressive symptoms (Liu et al., 2016; Riemslagh et al., 2021). Some fail to replicate motor phenotypes at all despite the presence of RNA foci and DPRs (O'Rourke et al., 2015). The factors influencing these dramatic changes in phenotype, either genetic or environmental, remain unknown. It is possible that other processes, possibly linked to RNA foci or DPR formation, play a bigger role in C9orf72‐related motor neuron cell death than degeneration at the NMJ. However, even these pathological hallmarks of C9orf72‐linked ALS remain controversial, since O'Rourke et al. showed RNA foci and DPR aggregation did not influence motor behaviour, while in other models, DPR formation, but no RNA foci, is present along with a severe motor phenotype (Riemslagh et al., 2021). As such, these C9orf72 models still fail to recapitulate the ALS pathology seen in patients. However, it could be argued that there is not yet a complete C9orf72 model of ALS, since the largest repeat expansion encompasses 500 repeats, whereas those in patients can be in the thousands (Liu et al., 2016).

Different muscle groups, and the motor neurons that innervate them, are known to have varying degrees of vulnerability in ALS. Specifically, fast‐twitch muscle groups are known to be affected at earlier time points compared to slow‐twitch muscles in the SOD1^G93A^ mouse (Gould et al., 2006; Vinsant et al., 2013). However, the reasoning for this differential vulnerability remains unclear. As highlighted throughout this review, different studies have analysed a variety of different muscle groups, some only fast‐twitch muscles and some only slow‐twitch muscles, making direct comparison between patterns of denervation difficult. Additionally, mature NMJs in non‐diseased mice are known to be morphologically different across distinct muscles (Mech et al., 2020). Furthermore, full comparative studies across different muscle groups over consecutive disease time points are lacking in most other mouse models of ALS, and would help to elucidate the mechanisms and perhaps any differences in patterns of vulnerability across ALS mouse models. Relatedly, molecular signatures of the extraocular muscles, given their resistance to ALS progression, may yet provide further therapeutic opportunities. Additional information regarding NMJ degeneration in ALS disease progression may come from work done in other animal models such as rat (Dong et al., 2020), *Drosophila* (Kankel et al., 2020) or zebrafish (Shaw et al., 2018).

Every other feature of ALS, from site of clinical onset to genetic background to molecular pathology, is extremely heterogeneous. Early NMJ dysfunction seems to be a commonality across different types of ALS and is therefore an important early disease readout and a druggable target relevant to most, if not all patients. Further analysis of the role of NMJ dysfunction in the newer mouse models of ALS will provide more evidence of its role in early pathogenesis. Given the heterogeneity of ALS, a more comprehensive NMJ analysis is desirable and could be achieved through using standardised muscles in analysis, for example the fast‐twitch TA versus the slow‐twitch *soleus*, and by utilising aNMJ‐morph, a semi‐automated Fiji macro for NMJ analysis. The power of aNMJ‐morph lies within its yield of 20 morphometric variables that allow for differentiation of subtle differences (e.g., branching, fragmentation or size variables) between disease models and consistency across research groups (Minty et al., 2020). However, caution should be taken when translating findings at the NMJ from mouse models into humans. While the mouse NMJ shows a characteristic ‘pretzel‐like’ shape, the human NMJ is more fragmented in healthy subjects, as shown in Figure 1, and appears to be remarkably stable throughout the healthy ageing process compared to the mouse NMJ (Jones et al., 2017). Additionally, some NMJ phenotypes in mouse models fail to translate to human samples, for example, in cancer cachexia where there was no evidence of NMJ changes in human patient samples despite clear phenotypes in mouse models (Boehm et al., 2020). Bearing in mind the body of evidence for NMJ pathology in ALS patient samples, it is likely to be a true ALS phenotype, but these discrepancies should be considered. Through combining our knowledge across animal models and into ALS patients, NMJ degeneration has the opportunity to provide both an early disease readout and a therapeutic target in future research.

## CONFLICT OF INTEREST

None.

## AUTHOR CONTRIBUTIONS

AA, IB and HC wrote, revised and edited this manuscript.
